# Exploring the potential of NTME/GC-MS, in the establishment of urinary volatomic profiles. Lung cancer patients as case study

**DOI:** 10.1038/s41598-018-31380-y

**Published:** 2018-08-30

**Authors:** Priscilla Porto-Figueira, Jorge Pereira, Wolfram Miekisch, José S. Câmara

**Affiliations:** 1 CQM - Centro de Química da Madeira, Universidade da Madeira, Campus Universitário da Penteada, 9020-105 Funchal, Portugal; 2 Department of Anaesthesiology and Intensive Care Medicine, Rostock Medical Breath Research Analytics and Technologies (ROMBAT), University Medicine Rostock, Rostock, Germany; 3 Faculdade de Ciências Exatas e da Engenharia da Universidade da Madeira, Campus Universitário da Penteada, 9020-105 Funchal, Portugal

## Abstract

The growing cancer incidence and mortality worldwide claims for the development of novel diagnostic strategies. In this study we aimed to explore the potential of an innovative methodology, based on a needle trap microextraction (NTME), combined with gas chromatography-mass spectrometry (GC-MS), as new approach to isolate and profile urinary volatile organic metabolites (VOMs) from lung cancer (LC) patients and healthy individuals (CTRL). In this context, different experimental parameters with influence of NTME extraction efficiency including, temperature, equilibration time, headspace volume, ionic strength, pH, effects of sample volume and stirring, were investigated and optimized. For the DVB/CarX/Car1000 needle trap device (NTD), the best results were obtained using 40 mL headspace of a 4-mL acidified (pH = 2) urine sample with 20% NaCl and an extraction temperature of 50 °C for 40 min of equilibration time. The stability of the isolated VOMs was investigated up to 72 h after extraction. From the VOMs identified, belonging namely to ketones, sulphur and benzene derivatives, 98 presented a frequency of occurrence above 90%. Data were processed by discriminant analysis, retrieving differentiated clusters for LC and CTRL groups. As far we are aware, this is the first study using NTME/GC-MS to establish urinary volatomic profiles. Preliminary results are very promising, as broad and comprehensive volatile profiles were obtained. Moreover, the extended storage stability of the NTD devices opens new opportunities for sampling other matrices in a wide range of applications.

## Introduction

**Figure 1:**
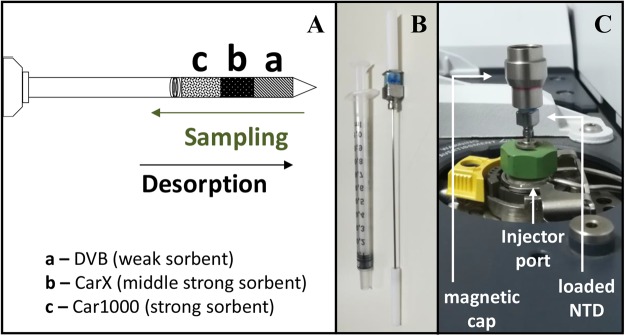
Configuration of the Needle Trap Device (NTD) used in this work. (**A**) Schematic representation of the triple bed DVB/CarX/Car1000 NTD, which allows the sequential retention from the big and polar to the small and less polar volatiles (DVB, divinylbenzene, is a week sorbent that retains mainly C7 to C20 compounds; CarX, Carboxen X, is a middle strong sorbent with higher affinity for C3 to C8 volatiles; Car1000, Carbopack 1000, is the strongest sorbent, retaining mainly C2 to C6 compounds). (**B**) The experimental layout used, (**C**) the DVB/CarX/Car1000 NTD loading the extracted volatiles in the GC-MS.

According to the most recent statistic available data (WHO, 2012), cancer was the second leading cause of death from non-communicable diseases worldwide, accounting for 8.2 million deaths^1^. The increase of the disease is overwhelming, with 3 million new cases reported every year^1^. This has been possible because conventional diagnostic methods for cancer have high costs and most of them are also quite invasive, causing mental and physical discomfort to the patient and even some risks for his life. For these reasons, demand for non-invasive strategies, able to obtain early information, less expensive and more amenable to the patients are essential to improve long term survival rates and quality of life. The characterization of the human metabolome opens new diagnosis opportunities, particularly in the identification of potential new cancer biomarkers using non-invasive methodologies targeted for early diagnosis. The use of volatile organic metabolites (VOMs) can be useful for this purpose, as well for monitoring the disease throughout its development, recurrence or treatment^2,3^. Biological fluids such us urine are rich in volatile compounds and this property has been used as a means of diagnosis, at least, since the ancient Greeks Hippocrates and Galen correlated the urinary odour with diabetes and kidney failure^4^. Theoretically, urinary volatomic profiles could be useful to characterize the disease itself as well as disease progression or response to therapy. In this context, VOMs identified in biological fluids, including urine, saliva and exhaled breath, constitute a very useful tool with potential to discriminate several diseases, including cancer^2,5,6^. However, due to the low concentration of most VOMs in the biofluids, the use of highly efficient extraction and analytical techniques able to isolate, identify and quantify these human metabolites, even at trace levels, is crucial for the success of such approach. Currently, methodologies as solid phase microextraction (SPME) combined with gas chromatography-mass spectrometry (GC-MS) analysis, deliver reliable and reproducible VOMs profiles of different biological samples^2,5–7^ but simultaneously the availability of extraction materials and basic equilibration mechanisms make them very selective. Silva *et al*.^5,6^ demonstrated the potential of certain urinary VOMs to discriminate different types of cancer, including breast^5^ and colon^6^ cancer. Matsumura *et al*.^8^ also established differences between the urinary profiles of lung cancer (LC) model mice with mices without any disease. Needle trap microextraction (NTME) is an innovative alternative to the common equilibration-based microextraction methodologies used to obtain volatile fingerprints from different matrices. NTME combines the advantages offered by SPME and solid phase extraction (SPE)^9^, with robustness, by employing resistant needles, also known as Needle Trap Devices (NTDs). NTDs can be adapted to the properties of the target volatiles by means of commercially available sorbent materials, particularly their affinity to the sorbent used (Fig. 1)^10^. Similarly to SPME, the technique requires small sample amounts, but the sensitivity of the methodology can be increased due to its exhaustive working mode^10^.

The objective of this work was to evaluate the potential of an innovative extraction methodology, NTME, combined with GC-MS and multivariate statistical analysis (MVSA) to establish the urinary volatomic patterns from LC patients and healthy subjects (CTRL) as a powerful platform to discriminate among LC and CTRL groups and identify putative volatile biomarkers. Different experimental parameters influencing the NTME efficiency, namely the equilibrium time, temperature, headspace volume, ionic strength, sample volume and sample agitation, were investigated and optimized. In addition, the stability (time of storage) of the extracted VOMs in the NTDs was also evaluated.

## Results and Discussion

### Optimization of NTME Extraction Parameters

To improve the efficiency of the NTME for the isolation of volatiles from headspace over urine samples, an univariate optimization strategy was applied to select the best conditions for a feasible and reliable experimental protocol. This approach follows previous work involving VOMs extraction from different biological matrices using SPME^6,7,10^. Accordingly, seven experimental parameters, sample volume, pH and ionic strength (% NaCl), temperature, headspace volume, equilibration time and stirring, were evaluated and optimized. The NTD configuration used was custom made triple layered DVB/CarX/Car1000 (Shinwa Ltd. Japan - Fig. 1), which was previously shown to be particularly tailored for applications aiming a comprehensive volatile characterization of low volume, water-saturated samples^10^.

### Sample pH

**Figure 2:**
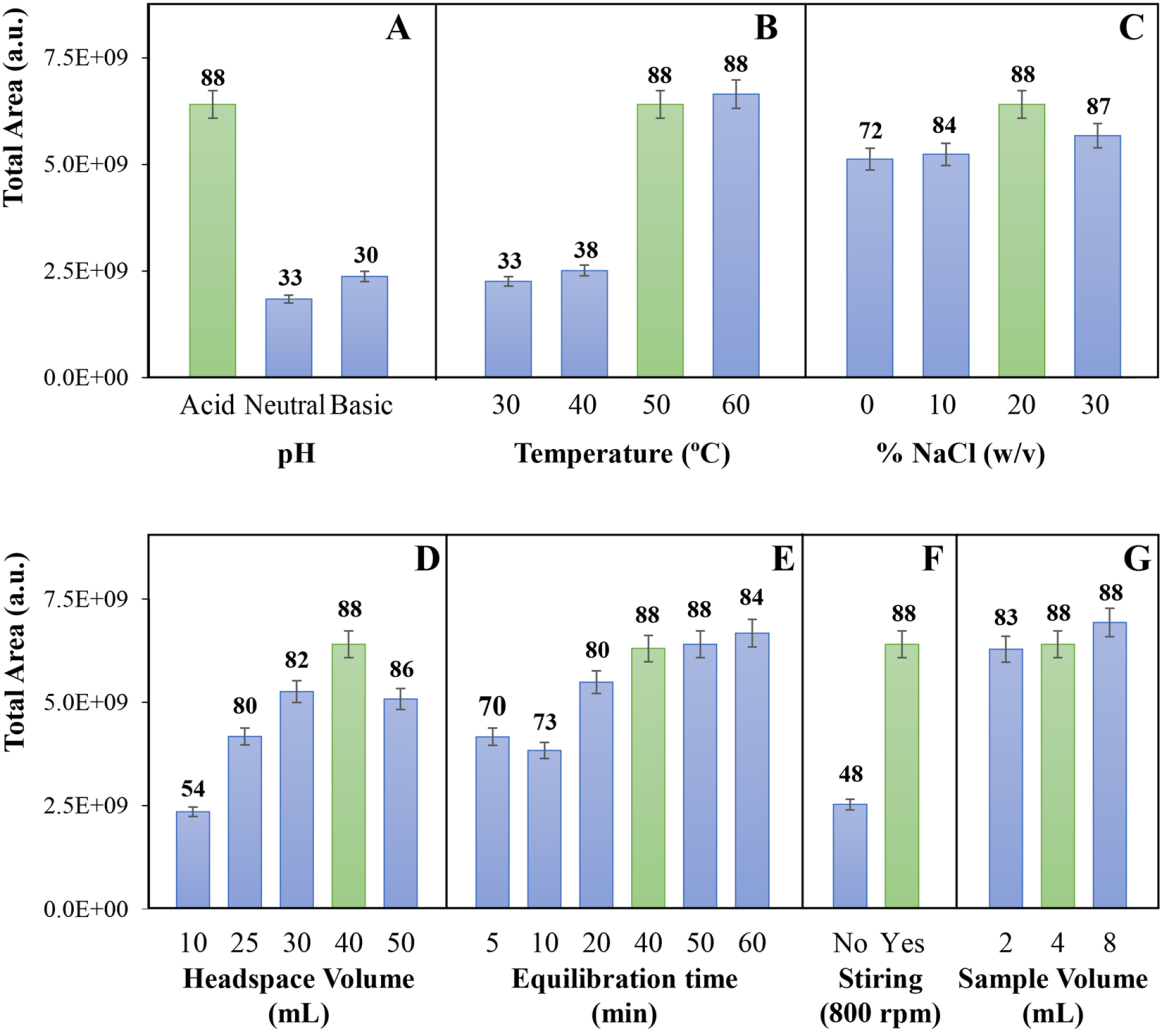
Optimization of different parameters affecting NTME: pH (**A**), extraction temperature (**B**), ionic strength through NaCl concentration (**C**), volume of the sample headspace (**D**), equilibration time (**E**), sample stirring (**F**) and volume (**G**). The number of VOMs identified in each experimental condition is also indicated in the top of each bar.

Previous studies have shown that the VOMs present in urine are mostly acidic^5,6,11^. Thus, the extraction efficiency will be greater if the metabolites are in their protonated form. On the other hand, many metabolites are eliminated through urine in conjugated forms, being also characterized by their low volatility and hydrophilic nature^12^. Rocha *et al*.^11^ have shown both the number of VOMs present in urine, as well as their associated relative areas, were higher in acidic conditions. In this work, we verified that urine acidification corresponds to an increase in the VOMs extraction efficiency, both in the number of volatiles identified, as well as in terms of total peak area (Fig. 2A). These results are therefore very interesting and explained by the polar nature of the urinary volatiles which, in the protonated form, may diffuse more easily from the sample to the headspace. It is important to note that all the VOMs identified in the basified (using NaOH) and neutralized (using NaOH or HCl) samples, pH 11 and 7, respectively, were also found in the acidified (using HCl) urine samples (pH 2) with higher responses. The only exception to this observation was acetone (Supplementary Fig. 1). All subsequent extractions were therefore carried out using acidified urine samples (pH 2).

### Extraction Temperature

The extraction temperature affects the extraction efficiency as higher temperatures facilitate the mass transfer of the compounds from the bulk sample to the headspace vapor phase. This increases the diffusion coefficient and decreases the distribution constant, reaching faster the balance between the two phases^13,14^. By increasing the temperature, we therefore can reduce the extraction time. This, however, is only valid until thermal degradation and/or isomerization of the sample metabolites occurs^13^. We have assayed extractions at 30 °C, 40 °C, 50 °C and 60 °C and the results shows a considerable increase in terms of number of extracted compounds and total area from 30 °C up to 50 °C, but not a relevant gain from 50 °C to 60 °C (Fig. 2B). This result agrees with our previous work using other extraction methodologies for the characterization of urinary volatile profiles^5,6^. Based on the obtained results, 50 °C was selected as the most appropriate temperature for the extraction of urinary volatile metabolites.

### Ionic Strength

The addition of salt (NaCl) to urine samples increases the ionic strength, enhancing the salting-out effect during the extractive process and therefore facilitating the diffusion of metabolites to the vapor phase^12,13^. On the other hand, excess of salts in solution do not favour the extraction^13^. In fact, we obtained a gain in the extraction efficiency with ionic strengths up to 20% NaCl (w /v). At higher NaCl concentrations (30% NaCl; w/v), a slight decrease in the total area was observed (Fig. 2C), although particular VOMs, such as dimethylsulfide and isoprene, have better responses under higher ionic strengths (Supplementary Fig. 1). Considering the total area, a concentration of 20% NaCl (w/v) was selected for the extraction of urinary VOMs.

### Sampling Headspace

Since NTME is an exhaustive technique, the sample headspace volume that is loaded through the sorbent is proportional to the response until the sorbent saturation is reached (breakpoint). Thus, by increasing the headspace volume of the sample, we will increase the extraction efficiency. In the evaluation of this parameter, the equilibration time and sample flow are also important parameters and should be considered. The first of these interfering factors was assayed in this work, while the second, the sample flow, was used as described by Trefz *et al*.^15^, that previously optimized this condition for the exhaled breath analysis using NTME^15^. Also in that report, the authors demonstrated that the extractive response of the sorbent was greater when larger sample headspace volumes were used, at least for the model metabolites isoprene, pentane, toluene and pentanal^15^. Furthermore, the breakthrough volume of this type of NTD was not reached up to a headspace volume of 60 mL^15^ and a linear response was obtained in this range. Our results show a considerable increase in the number and total area of the VOMs extracted between 10 and 30 mL of headspace, whereas the gain is very moderate up to 40 mL of sample headspace and there is a decrease in the total area of the metabolites extracted (and less 2 VOMs identified) for 50 mL of headspace (Fig. 2D). Based on the obtained results, and in order to minimize the extraction time and increase the reusability of the sorbents, 30 mL was selected as the appropriate headspace sample volume.

### Equilibration Time

The equilibration time of the urine sample with its headspace affects the VOMs diffusion to the vapor phase. Analysis of the results obtained shows an incremental response of the total area and number of metabolites up to 40 min of equilibration (Fig. 2E). For a longer equilibration time of 60 min, the increase in the total area is not significant and the number of compounds decreases from 88 to 84. This suggests that some metabolites degradation may occurs as result of the prolonged time under heating and stirring conditions. Furthermore, the behaviour of specific VOMs analysed individually is not uniform, with better responses observed with 60 min of equilibration for (+) - 4-carene and dimethylsulfide, the opposite for acetone and 2-butanone and irregular variations for toluene, phenol, isoprene and 2-pentanone (Supplementary Fig. 1). An equilibration time of 40 min was therefore selected as the most favourable.

### Sample Stirring

Constant and efficient sample stirring facilitates sample homogenization^16^ and VOMs difusion from the liquid to the vapor phase, thus reaching equilibrium more quickly^13^. Our results show that a constant sample stirring at 800 rpm contributes to a considerable improvement in the extraction efficiency of the methodology by comparison with a no stirring approach. This is clearly expressed by the large increase (almost two times) in the number of extracted metabolites and total area (Fig. 2F). An equivalent result was obtained for several metabolites analysed individually (data not shown).

### Sample Volume

In the evaluation of the optimum sample volume, we assayed 2, 4 and 8 mL of urine. The results obtained reveals a small increase in the total area and number of VOMs identified from 2 to 4 mL, but no significant gain with 8 mL samples (Fig. 2G). When this analysis was detailed to specific VOMs, heterogeneous variations were obtained for the selected metabolites (Supplementary Fig. 1). Considering these results, particularly the negligent gain in the extraction efficiency by doubling the sample volume from 4 mL to 8 mL, the lower sample volume was selected as the most suitable for the methodology being optimized. This selection also allows a significant reduction in the sample residues produced.

### Storage Time

**Figure 3:**
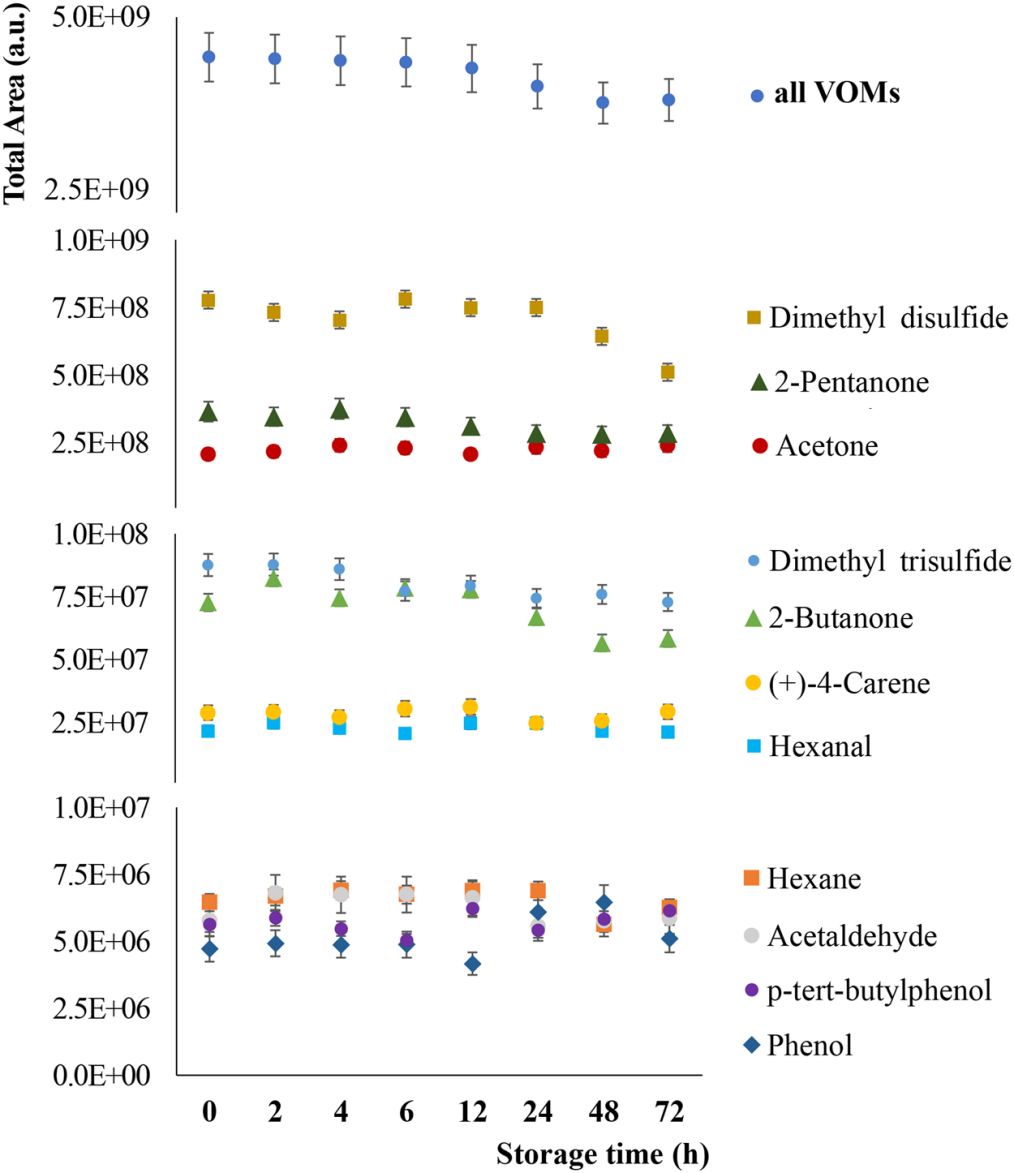
Analysis of the VOMs storage stability in the triple NTD up to 72 h upon extraction.

The stability of the extracted VOMs in the sorbent as a function of the storage time (0–72 h) was also evaluated. This parameter is very relevant because samples are collected outside the laboratory, mostly in clinical facilities, where patients are observed. In this context, the possibility of performing *in situ* sampling and store the extracted analytes in the NTDs for later analysis, therefore avoiding the transport of biological samples from the collection point to the lab, would be a major advantage. The results obtained were very interesting as we verified that during the first 12 hours of storage there are no significant differences (*p* < 0.05) in terms of total areas, while after 24 hours, the decrease in the metabolite stability is marginal (decrease of less than 10% total area compared to the starting point - VOMs extracted and analysed after sample collection). Even after 48 and 72 hours of extraction, the loss of extracted analytes is about 15% over the initial period, revelling therefore a high stability and reliability in the use of the NTDs as a sampling and storage device for the VOMs extracted at least up to 3 days after extraction (Fig. 3). This study was further extended to the analysis of a set of representative VOMs. As can be observed in Fig. 3, the decrease in total areas obtained for the selected metabolites is not appreciable, confirming the high stability of the selected metabolites in the sorbent during the period considered.

### VOMs Profile of Lung Cancer Patients

The urinary VOMs profile of LC was established using NTME followed by high-resolution GC-MS to process 30 urine samples from healthy subjects (CTRL) and 17 urine samples from LC patients. The typical chromatograms of the volatile urine profile of subjects from each study group is presented in the Supplementary Fig. 2.

The analysis of the chromatograms obtained allowed the identification of 98 VOMs with a frequency of occurrence (FO) higher than 90% in the volatile composition of the urine of all samples used in this study. Table 1 summarizes the metabolites identified for each group, the chromatographic data and the possible origin^17^.

**Table 1:** VOMs identified in urine samples from LC and CTRL groups by NTME/GC–MS methodology and respective possible origin (End - VOM endogenously produced, Ex – VOM resulting from exogenous sources, as diet (F), microbial (M), drug metabolism (D), environmental contamination (E), according to the data available in the human metabolome database^17^, Unkn – VOM whose origin is currently unknown).

	VOMs	RT (min)	KI _Exp_	KI _Lit_	LC		CTRL		Possible Orign
					FO	Total Area	FO	Total Area	
1	Pentane	4.38	660	650	82	9.3E + 04	100	6.3E + 04	End/Ex
2	Methyl chloride	4.49	671	—	71	7.6E + 05	100	1.5E + 05	End/Ex
3	Hexane	4.54	676	600	100	7.5E + 04	97	4.9E + 04	End/Ex
4	Ethyl ether	4.60	682	640	94	1.7E + 04	87	1.3E + 04	Unkn
5	Isoprene	4.67	689	633	100	9.4E + 05	100	8.0E + 05	End
6	Methanethiol	4.82	703	702	100	1.6E + 06	100	1.3E + 06	End
7	Acetaldehyde	4.85	706	703	88	3.1E + 06	97	4.9E + 05	End/Ex(DM)
8	Carbon disulfide	5.08	726	723	100	1.1E + 06	100	2.0E + 05	Ex
9	Dimethyl sulfide	5.21	737	737	94	6.3E + 04	100	8.2E + 04	End/Ex(DM)
10	*trans*-2-Methyl-1,3-pentadiene	5.45	757	760	100	9.4E + 04	83	1.4E + 04	Unkn
11	Furan	5.59	768	760	100	1.9E + 07	100	2.0E + 07	End
12	Acetone	5.84	788	785	100	2.6E + 07	100	3.7E + 07	End(M)
13	Tetrahydro-2,2,5,5-tetramethylfuran	6.42	830	—	100	5.2E + 05	93	4.6E + 06	Unkn
14	2-Methylfuran	6.59	842	843	100	3.2E + 06	100	3.0E + 06	End
15	3-Methylfuran	7.12	876	877	82	5.3E + 05	97	2.0E + 06	Ex(F)
16	2-Butanone	7.20	881	881	94	3.8E + 06	100	5.1E + 06	End
17	3-Methyl-2-butanone	7.90	915	918	100	8.5E + 05	90	1.4E + 06	End
18	Benzene	8.22	927	924	100	6.3E + 04	80	2.8E + 05	Ex(E)
19	2,5-Dimethylfuran	8.59	940	943	100	5.5E + 05	80	6.7E + 05	Ex(F)
20	2-Ethylfuran	8.62	941	941	100	7.0E + 05	70	3.8E + 05	Ex(F)
21	1,6-Dimethylhepta-1,3,5-triene	8.71	944	—	100	4.1E + 05	93	2.3E + 05	Unkn
22	1,2,5,5-Tetramethyl-1,3-cyclopentadiene	8.99	953	—	100	2.0E + 05	100	1.5E + 05	Unkn
23	5-tert-Butyl-1,3-cyclopentadiene	8.93	951	—	94	4.0E + 05	63	3.1E + 05	End/Ex(F)
24	2-Pentanone	9.37	966	969	100	1.4E + 07	100	1.7E + 07	Ex(F)
25	2,4-Dimethyl-3-pentanone	10.15	989	995	100	4.7E + 06	100	1.3E + 06	End/Ex(F)
26	2-Hexanone	11.10	1001	1082	100	9.0E + 05	100	4.9E + 05	End
27	3,3-Dimethyl-6-methylenecyclohexene	10.78	1006	—	100	1.6E + 05	93	9.7E + 04	Unkn
28	3-Methyl-2-pentanone	10.91	1008	1005	100	1.2E + 06	100	1.1E + 06	End/Ex(F)
29	Thiophene	11.25	1016	1017	100	1.9E + 05	100	2.4E + 05	End/Ex (F)
30	1,3-Dimethyl-1-cyclohexene	11.66	1024	—	100	9.5E + 05	100	8.8E + 05	Unkn
31	Toluene	12.06	1032	1033	100	8.7E + 05	100	2.1E + 06	Ex(PF)
32	3-Hexanone	12.71	1044	1047	100	7.1E + 05	100	1.7E + 06	End
33	2-Methyl-3-hexanone	12.93	1044	—	94	5.6E + 05	60	5.9E + 05	Unkn
34	2-Ethyl-4-methylimidazole	12.97	1049	—	100	1.5E + 05	100	2.4E + 05	Unkn
35	Dimethyl disulfide	13.90	1065	1065	100	9.8E + 07	100	1.0E + 08	End
36	Hexanal	14.52	1075	1075	100	9.3E + 05	93	3.4E + 05	End
37	2-Methylthiophene	14.99	1083	1085	100	1.7E + 05	97	1.9E + 05	Ex(F)
38	2-Methyl-(E)-2-butenal	15.41	1089	1088	100	1.4E + 05	83	1.0E + 05	End/Ex(F)
39	6,6-Dimethylhepta-2,4-diene	15.53	1091	—	100	1.3E + 05	93	7.1E + 04	Unkn
40	2-Ethyl-5-methylfuran	15.64	1092	1034	94	7.8E + 05	73	9.0E + 04	End/Ex(F)
41	2,2,6-Trimethyl-6-vinyltetrahydropyran	15.83	1095	1095	100	1.4E + 05	100	2.5E + 05	End/Ex(F)
42	7,7-Dimethyl-9-oxatricyclo[6.2.2.0(1,6)]dodecan-10-one	17.21	1114	—	94	1.5E + 05	93	1.2E + 05	Unkn
43	4-Heptanone	17.42	1117	1118	100	5.0E + 07	100	3.0E + 07	End
44	p-Xylene	18.05	1124	1123	100	1.1E + 05	100	2.4E + 05	Unkn
45	1,5,5-Trimethyl-6-methylene-cyclohexene	18.21	1126	—	100	1.7E + 05	100	1.6E + 05	Unkn
46	Ethyl methyl disulfide	18.51	1130	1141	94	6.5E + 04	97	5.3E + 04	Ex(F)
47	α-Phellandrene	19.21	1138	1143	76	3.0E + 04	90	9.5E + 04	End/Ex(F)
48	3-Heptanone	19.60	1143	1148	100	6.6E + 04	97	9.3E + 04	End/Ex(F)
49	α-Terpinene	20.27	1150	1159	100	7.1E + 05	100	2.4E + 06	End/Ex(F)
50	1,4-Cineole	20.80	1156	1164	100	4.2E + 05	100	1.2E + 06	End/Ex(F)
51	2-Heptanone	22.37	1172	1173	100	1.0E + 06	100	4.8E + 05	End
52	Eucalyptol	23.33	1181	1183	82	2.7E + 05	97	4.7E + 05	Ex(D)
53	1-Ethyl-3-methylbenzene	24.54	1192	1200	88	9.2E + 04	90	2.1E + 05	Unkn
54	2-Pentylfuran	25.55	1201	1203	100	8.7E + 05	97	4.9E + 05	End/Ex(F)
55	γ-Terpinene	25.88	1205	1213	100	3.1E + 05	100	1.2E + 06	Ex(F)
56	3,4-Dimethylthiophene	28.15	1226	1240	100	3.7E + 05	100	1.2E + 06	Ex(F)
57	o-Cymene	28.99	1234	1248	100	1.3E + 07	100	7.0E + 07	Ex(F)
58	Isoterpinolene	29.87	1242	1270	65	1.5E + 05	97	8.0E + 05	End/Ex(F)
59	Methyl allyl disulfide	31.13	1253	1253	100	4.6E + 06	100	2.8E + 06	Ex(F)
60	1,3-Dithiane	31.64	1257	1296	100	6.5E + 05	100	8.9E + 05	End/Ex(F)
61	1,3-Dimethyl-2-ethylbenzene	34.74	1281	1347	100	9.5E + 06	83	1.5E + 07	Unkn
62	2,2,6-Trimethylcyclohexanone	35.21	1284	1284	100	8.9E + 04	93	8.7E + 04	End/Ex(F)
63	1,2,3-Trimethylbenzene	36.63	1295	1295	100	3.3E + 05	100	4.4E + 05	Unkn
64	2-methoxy-5-methyl-Thiophene	39.71	1321	—	100	1.4E + 05	93	7.7E + 04	Unkn
65	Dimethyl trisulfide	42.37	1343	1343	100	7.5E + 06	100	8.4E + 06	End/Ex(M)
66	2-Methyl-5-(methylthio)furan	44.17	1358	—	100	1.1E + 06	97	5.9E + 05	Ex(F)
67	Isophorone	46.55	1376	1541	100	5.5E + 05	93	1.7E + 05	End/Ex(F)
68	2,3-Dihydro-1,1,5,6-tetramethyl-1H-indene	48.96	1393	—	82	9.9E + 05	100	1.1E + 06	Unkn
69	1,1,6,7-Tetramethyl-Indan	49.61	1398	—	65	7.1E + 05	93	1.1E + 06	Unkn
70	p-Cymenene	50.24	1400	—	100	4.4E + 06	97	1.8E + 07	End/Ex(F)
71	Durene	55.67	1449	1446	94	5.8E + 05	90	1.4E + 06	Unkn
72	Acetic acid	58.07	1468	1465	94	1.4E + 06	93	1.5E + 06	End/Ex(DM)
73	1,2,5,5,6,7-Hexamethylbicyclo[4.1.0]hept-2-en-4-one	60.25	1484	—	94	4.5E + 05	100	9.6E + 05	Unkn
74	1-(4-Methoxyphenyl)-1,3-butanedione	60.57	1487	—	100	2.7E + 05	100	5.4E + 05	Unkn
75	2-Ethyl-1-hexanol	61.07	1490	1490	100	2.9E + 05	60	1.1E + 05	End/Ex(F)
76	m-Anisalcohol	61.85	1496	—	100	3.1E + 05	93	3.6E + 05	Ex(F)
77	2-Acetylfuran	61.98	1497	1498	100	3.7E + 05	90	4.6E + 05	Ex(F)
78	5-Methylfurfural	69.92	1565	1567	100	6.5E + 04	60	5.6E + 04	Ex(F)
79	trans-(-)-5-Methyl-3-(1-methylethenyl)-cyclohexene	69.91	1565	—	100	6.4E + 04	43	4.6E + 04	Unkn
80	1,1,4,5-tetramethyl-2,3-dihydro indene	75.43	1613	—	100	2.1E + 05	60	1.8E + 05	Unkn
81	1,1,3-Trimethyl indane	78.58	1648	—	100	1.3E + 05	70	1.7E + 05	Unkn
82	Dehydro-Ar-ionene	83.90	1706	1712	100	1.0E + 07	100	4.6E + 06	Ex(F)
83	α-Curcumene	87.38	1764	1764	71	6.5E + 04	100	3.0E + 06	Unkn
84	1-(2,6,6-Trimethyl-1,3-cyclohexadien-1-yl)-2-buten-1-one	89.92	1810	1801	94	1.9E + 05	63	1.8E + 05	End
85	1,1,3-Trimethyl-1H-indene	90.57	1829	—	94	2.5E + 05	77	7.0E + 05	Unkn
86	2,5,8-Trimethyl-1,2-dihydronaphthalene	90.88	1838	1999	94	5.9E + 05	33	3.3E + 05	Unkn
87	Guaiacol	91.91	1868	1868	100	5.6E + 05	100	4.4E + 05	End
88	α-Calacorene	93.21	1909	1910	100	6.6E + 05	100	1.5E + 06	End/Ex(F)
89	Phenol	95.12	2009	2009	100	2.1E + 06	100	1.2E + 06	End/Ex(E)
90	p-Cresol	96.37	2074	2074	100	1.5E + 07	100	1.1E + 07	End/Ex(M)
91	1-(2,3,6-Trimethylphenyl)-3-buten-2-one	97.02	2107	2180	100	3.5E + 05	80	2.6E + 05	Unkn
92	p-Ditolylmethane	98.25	2169	—	100	1.1E + 05	83	1.4E + 05	Unkn
93	Carvacrol	98.50	2182	2183	100	7.0E + 05	90	4.8E + 05	End/Ex(F)
94	4-tert-Butyl-2-Bromophenol	98.72	2193	—	100	4.1E + 05	97	1.9E + 05	End/Ex(F)
95	Cadalene	98.94	2204	2203	100	2.6E + 05	97	4.7E + 05	Unkn
96	4-tert-Butylphenol	99.83	2248	—	100	1.1E + 06	100	5.8E + 05	End/Ex(F)
97	3,5-Di-t-butylphenol	100.72	2256	2310	71	6.1E + 04	100	1.4E + 05	End
98	Benzoic Acid	102.75	2402	2405	76	1.4E + 05	97	1.2E + 05	Ex(FM)

RT- Retention time (min); KI – Kovats Index (Exp – experimental, Lit – theoretical KI values reported in literature). FO – Frequency of occurrence.

**Figure 4:**
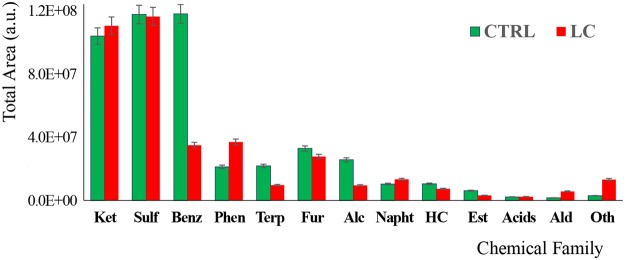
Characterization of the VOMs profiles obtained for LC and CTRL groups by chemical family. These simplified profiles were obtained using only the VOMs with a frequency of occurrence (FO) above 90%. Legend: Acids - Organic acids, Alc – Higher Alcohols, Ald - Aldehydes, Benz - Benzene derivatives, CTRL – control, Est – Esters, Fur – Furans, HC – Hydrocarbons, LC – patients with colon cancer, Napht - Naphthalene derivatives, Oth – Others, Phen – Phenols, Sulf – Organosulphurs, Terp - Terpene derivatives.

The metabolites identified in CTRL and LC urine samples span diverse chemical families, being the most representative the ketones, in addition to sulfuric, benzenic, furanic and terpenic compounds. Figure 4 shows the distribution of the metabolites identified in the groups under study according to their chemical families. As can be easily observed, the urinary profiles of both groups are dominated by sulphur compounds and ketones (each class representing nearly 30% of the total area), with the remaining classes representing less than 10% of the total area obtained in each group. The only exception are benzenes, which are highly abundant in the control samples (around 30% of the total area obtained), while in the patients group, only a third of the equivalent representativeness was found. In the same direction, terpenics and alcohols are significantly more abundant in the control group. Phenolics and aldehydes, in turn, are more abundant in the LC than in the control group. These volatomic differences are eventually related with the altered metabolic activity that cancers cells have in comparison with the normal cells. Nevertheless, external factors contributing with exogenous VOMs must be also considered as important interferent factors^5,6^. And this problem cannot be overcome just by discarding such metabolites, because for many of them it is not possible to associate a unique origin, being very difficult to completely elucidate their metabolic pathway^6,18,19^.

### Statistical Analysis

**Figure 5:**
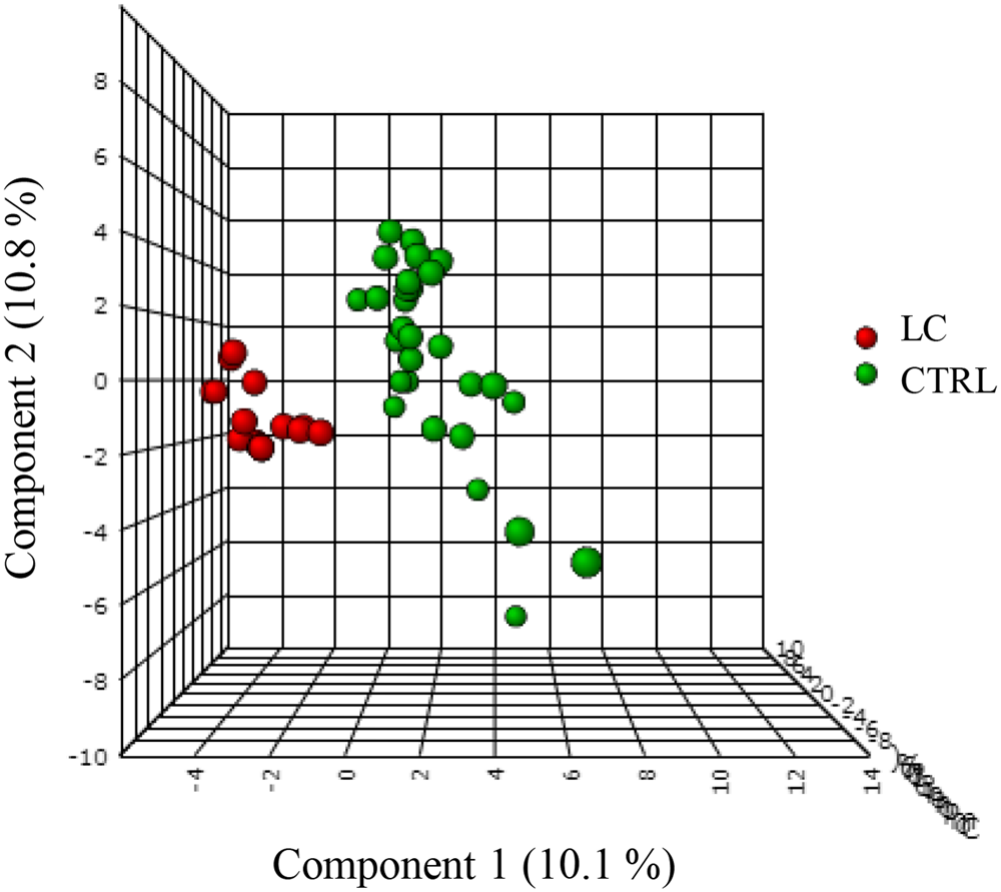
Application of PLS-DA to the experimental data obtained. Legend: CTRL – control samples, LC – lung cancer samples, PLS-DA - supervised partial least square discriminant analysis.

Following the characterization of the urinary volatile profiles of LC and CTRL subjects, the data obtained was processed with different statistical tools to assess the existence of discriminative models able to distinguish LC from CTRL samples. In addition to the procedures described in the experimental section, and to try to obtain more robust and meaningful models, only the VOMs identified with a FO of at least 90% were considered in the statistical analysis. Moreover, the VOMs unambiguously reported in the literature^17^ as exogenous were also discarded. Firstly, a variable normalization was applied to the data matrix to obtain a homogeneous distribution. This procedure reduced the size of the data matrix by eliminating redundant variables that do not contribute to differentiate the groups being analysed. Following this, a multivariate analysis using supervised partial least square discriminant analysis (PLS-DA) models^20,21^ was performed with the MetaboAnalyst 3.0 web based tool^22^. The statistical model obtained segregates LC an CTRL samples in two clusters corresponding to the cancer and non-cancer samples analysed (Fig. 5). Additionally, top 29 VOMs differentially expressed were found with variable importance in projection (VIP) > 1 (Table 2). Among these, several VOMs have been previously involved in the discrimination of different clinical conditions. Hexanal, as well as other aldehydes, for instance, has been already reported by several authors as discriminant metabolites for LC^23–25^. In turn, Rudnicka *et al*. showed that, although carbon disulfide was present in the volatile profile of healthy individuals and smokers, it has a higher significance in LC, with a considerably higher response in the patients^26^. A similar result was reported by Buszewski *et al*.^27^ when comparing the volatile composition of normal and stomach cancer tissue. Regarding 2-heptanone, Hanai *et al*.^28^ identified this ketone in the urine and culture of rats LC cell lines, with a higher abundance than the respective control groups. Finally, to check the robustness of statistic model obtained, a random permutation test with 100 permutations was performed with PLS-DA model. The permutation test yielded a R2 (Goodness of fit) of 0.915 and a Q2 (Predictive ability) of 0.760, indicating that the model is not over fitted and has good predictive ability to distinguish between the studied groups. It should be highlighted, however, that this is just a proof of concept study involving a limited number of samples. To be able to extrapolate the discrimination power for LC diagnosis, broader and extensive studies with a statistical significant number of samples and independent patient cohorts are mandatory. The methodology reported here constitutes a breakthrough given the superior sampling and storage abilities of the NTDs and the broader coverage of the volatomic profiles obtained and may therefore help to reach our ambitious aims.

**Table 2:** List of VIP VOMs obtained following PLS-DA analysis (score >1).

VOMs	Comp. 1	Comp. 2	Comp. 3
Methyl chloride	1.10	1.03	1.00
Acetaldehyde	1.34	1.33	1.34
Carbon disulfide	1.34	1.25	1.21
Dimethyl sulfide	1.19	1.16	1.19
Acetone	0.86	0.92	1.00
2-Butanone	0.99	0.95	0.99
1,6-Dimethylhepta-1,3,5-triene	1.77	1.66	1.64
2,4-Dimethyl-3-pentanone	1.11	1.04	1.03
3,3-Dimethyl-6-methylenecyclohexene	1.64	1.52	1.50
Thiophene	1.09	1.10	1.10
3-Hexanone	1.17	1.18	1.18
Hexanal	1.02	0.96	0.96
2-Ethyl-5-methylfuran	1.17	1.14	1.11
2,2,6-Trimethyl-6-vinyltetrahydropyran	1.31	1.34	1.30
α-Phellandrene	1.73	1.64	1.61
α-Terpinene	1.56	1.52	1.49
1,4-Cineole	1.11	1.14	1.13
2-Heptanone	1.06	1.04	1.03
1-Ethyl-3-methylbenzene	1.60	1.49	1.48
Isoterpinolene	1.47	1.48	1.46
1,2,3-Trimethylbenzene	1.17	1.16	1.13
2,3-dihydro-1,1,5,6-tetramethyl-1H-indene	1.68	1.59	1.59
α-Curcumene	1.50	1.40	1.37
1,1,3-trimethyl-1H-indene	1.17	1.25	1.23
α-Calacorene	1.26	1.28	1.26
p-Cresol	1.65	1.56	1.52
Carvacrol	1.19	1.17	1.22
4-tert-Butylphenol	1.68	1.56	1.52
3,5-Di-t-butylphenol	2.16	2.21	2.15

Comp. 1 – Component 1; Comp. 2 – Component 2; Comp. 3 – Component 3.

## Conclusions

To the best of our knowledge, this is the first study reporting the high throughput potential of NTME combined to GC-MS to characterize the volatomic profile of urine samples without complicated pre-treatments, so could be a versatile tool for future application, providing a good technical advance. In this context, different key parameters of the extraction procedure were evaluated and optimized according to the nature of the sample and its volatile composition. The optimized experimental layout involves a DVB/CarX/Car1000 NTD to extract 40 mL headspace of a 4-mL acidified urine added NaCl 20% (m/v), during 40 min at temperature of 50 ± 1 °C. The methodology was applied to 47 urine samples (30 CTRL samples and 17 LC samples), and a good dicrimination between both groups based in the volatomic profiles was obtained upon application of MVSA to the data matrix. This proof of concept study demonstrates the advantages of NTME/GC-MS in the characterization of complex urinary volatile profiles in addition to the potential of the methodology as useful platform towards the non-invasive disease diagnosis.

## Materials and Methods

### Chemicals and Materials

Hydrochloric acid (HCl), sodium chloride (NaCl), all the standards used for VOMs confirmation (purity higher than 98.5%) and the *n*-alkanes mixture containing C_8_–C_20_ straight-chain alkanes in hexane were obtained from Sigma-Aldrich (St. Louis, MO, USA). Helium, ultra-pure grade (Air Liquide, Portugal) was used as carrier gas in the GC system. Clear glass screw cap vials for extraction with PTFE/silica septa were purchased from Supelco (Bellefonte, PA, USA). The NTDs used in this work, “NeedleEx”, were custom manufactured by Shinwa Ltd., Japan (60 mm × 0:41 mm id, 0.72 mm od, triple bed configuration Divinylbenzene/Carboxen X/Carbopack 1000 - DVB/CarX/Car1000) and purchased from PAS Technology (Magdala, Germany). Prior to their use, NTDs were conditioned in a heating device (PAS Technology, Magdala, Germany) at 250 °C, under permanent helium flow for at least 20 h to eliminate any contaminations from the manufacturing process or shipping. Afterwards, both ends of the needles were sealed with Teflon caps and stored. Before being used, the NTDs were conditioned again for 30 min in the heating device.

### Subjects and Urine sampling

Thirty healthy individuals (14 males and 16 females, age = 18–65 years; control group – CTRL) without any known pathology recruited at the Blood Transfusion Medicine Service of Dr. Nélio Mendonça Hospital - HCF) and 17 LC patients (11 males and 6 females, age = 46–80 years, recruited at the Haemato-Oncology Unit of HCF), were randomly selected among the volunteers (Table 3). Participants were given sterile and disposable glass bottle collectors and instructed to collect the first urine in the morning after the rejection of the first urine stream. All participants were fully informed of the objectives of the study and signed the informed consent before donating the urine samples. After collection, each sample was individually homogenized, aliquoted in 5 mL ambar glass vials and stored at −80 °C until analysis. This study was previously approved by the Ethics Committee of HCF.

**Table 3:** Characterization of the groups of subjects recruited for this study, by diagnose, number of samples, gender, age and smoker habits, All the samples were collected after the approval by ethics committee of Hospital do Funchal, Madeira, Portugal.

#	Diagnose	Number of samples	Gender	Age (years)	Smokers
CTRL	Control	30	14 Male/16 Female	18–65	6
LC	Lung Cancer	17	11 Male/6 Female	46–80	0*

CTRL –Healthy individuals, LC – Lung Cancer patients, *All 17 subjects are ex-smokers.

### Optimization of NTME extraction

To increase the NTME efficiency, different key parameters were optimized according to the characteristics of the sample and extractive methodology^6,29^. This included (i) the pH of the sample (acid – pH 2, basic – pH 11, and neutral – pH 7), (ii) the extraction temperature (30 °C, 40 °C, 50 °C to 60 °C), (iii) the ionic strength (0, 10%, 20% and 30% NaCl, w/v), (iv) sample headspace volume (10, 25, 30, 40 and 50 mL), (v) the equilibration time (5, 10, 20, 40, 50 and 60 min), (vi) sample stirring (0 and 800 rpm) and (vii) sample volume (2, 4 and 8 mL) were tested using an univariate procedure to achieve extraction conditions suitable for this application (lowest sample volume, preferentially near room temperature and reasonable equilibration time). In this study, we also evaluated the storage time of the VOMs in the NTD (2, 4, 6, 12, 24, 48 and 72 hours after the NTME extraction). All extractions were performed in duplicate.

### NTME extraction

Following the optimization step, 4 mL of urine sample was insert in 20 mL tubes (extraction tubes - ETs) and added 0.5 mL HCl 5 M to acidify the sample (pH = 2). The pH was verified with the aid of a pH meter (Hanna Instruments) with a clean electrode. After the measurements, the electrode was carefully cleaned to avoid contamination between samples. To promote the salting-out effect, facilitating the transference of the urinary VOMs for the headspace, 20% NaCl (w/v) were added, the ET sealed, and the system equilibrated for 40 min at 50 ± 1 °C. Then, the NTDs pre-attached to a disposable 1 mL syringe were insert into the headspace of the ETs and 40 mL of the gas phase were manually loaded through the sorbent (40 withdraw-loading cycles, average speed 10 ± 2 mL/min). After the extraction, the syringe was discarded and the NTD was sealed in both ends with PTFE caps. Finally, the NTD was injected in the GC-MS at 250 °C for 30 seconds for thermal desorption of the extracted VOMs. Before the next extraction, the sorbent was reactivated by placing the NTDs in a conditioner at 250 °C under constant flow of helium (purity 5.0, Air Liquid, Portugal) at a constant pressure of 1 bar for 30 min.

### Gas chromatography-mass spectrometry analysis (GC-MS)

The analysis was carried out with an Agilent 6890N gas chromatograph system (Agilent Technologies, Palo Alto, CA, USA) coupled with an Agilent 5975 quadrupole inert mass selective detector. The separation of the extracted compounds was performed on a BP-20 fused silica capillary column (60 m × 0.25 mm I.D. × 0.25 µm film thickness). Splitless injection was employed using helium as carrier gas at a constant flow rate of 1.0 mL/min. Oven temperature conditions were: 45 °C (held for 2 min), followed by a gradient temperature ramp from 45 °C to 60 °C, held for 1 min at a rate of 0.7 °C/min, followed by a flow rate of 1.0 °C/min until 110 °C (held for 1 min); then a flow rate of 3.0 °C/min until 150 °C (held for 1 min) and finally from 150 °C to 220 °C held for 10 min at a rate of 15 °C/min. The injection and ion source temperatures were 250 °C and 230 °C, respectively. The mass spectra of the compounds were acquired in electron-impact (EI) mode at 70 eV. The electron multiplier was set by the auto tune procedure. Data acquisition was performed in scanning mode (mass range *m/z* = 35–300 amu; six scans per second). Chromatograms and spectra were recorded and processed using the Enhanced ChemStation software for GC-MS (Agilent Technologies, Palo Alto, CA, USA). VOMs identification was based on the comparison between the GC retention times (RT) of the chromatographic peaks with those, when available, of authentic standards run under the same conditions. MS fragmentation patterns were compared with those of pure compounds, and mass spectrum database search was performed using the National Institute of Standards and Technology (NIST) MS 05 spectral database. Finally, confirmation also involved the determination of the RI of each peak of C_8_–C_20_ *n*-alkanes series. Once again, the values were compared, when available, with values reported in the literature for similar chromatographic columns. Chromatographic peak areas, expressed in arbitrary units (a.u.) of area were determined using the FullScan chromatogram, and were used as an indirect approach to estimate the relative content of each volatile metabolite. For semi-quantification purposes, each sample was injected in triplicate, and the chromatographic peak areas (as kcounts amounts) were determined by a reconstructed full-scan chromatogram using for each compound some specific quantification ions: these corresponded to base ion (m/z 100% intensity), molecular ion (M^+^), and another characteristic ion for each molecule.

### Multivariate statistical analysis (MVSA)

In order to identify potential discriminative features for the selected groups (LC and CTRL), the statistical data analysis was carried out using the web based application, Metaboanalyst 3.0^22^. The data was normalized by the median, auto scaled and subjected to multivariate statistical analysis. Partial least squares-discriminant analysis (PLS-DA) was then used to visualize the separation between LC and CTRL groups and to identify set of VOMs able to discriminate between the two groups.

### Ethical approval

The study was approved by ethics committee of Hospital do Funchal, Madeira, Portugal.

### Informed consent

Prior informed consent was obtained from all the participants in the study with institutional review approval.

### Research involving human participants and/or animals

All protocols and procedures were adhered to institutional ethical standards and/or research committee and with the 1964 Helsinki declaration and its later amendments or comparable ethical standards.

## Electronic supplementary material

Supplementary Data

## References

[1] World Health Organization, W. H. O. Global status report on noncommunicable diseases 2014. (WHO Genebra, 2015). 10.1161/STROKEAHA.115.008097 25873596

[2] Broza YY, Zuri L, Haick H (2014). Combined volatolomics for monitoring of human body chemistry. Sci. Rep..

[3] Pereira J (2015). Breath analysis as a potential and non-invasive frontier in disease diagnosis: an overview. Metabolites.

[4] Bradley, M. *Smell and the ancient senses*. (Routledge, 2014).

[5] Silva CL, Passos M, Camara JS (2012). Solid phase microextraction, mass spectrometry and metabolomic approaches for detection of potential urinary cancer biomarkers–a powerful strategy for breast cancer diagnosis. Talanta.

[6] Silva CL, Passos M, Camara JS (2011). Investigation of urinary volatile organic metabolites as potential cancer biomarkers by solid-phase microextraction in combination with gas chromatography-mass spectrometry. Br. J. Cancer.

[7] Silva CL, Perestrelo R, Silva P, Tomas H, Camara JS (2017). Volatile metabolomic signature of human breast cancer cell lines. Sci. Rep..

[8] Matsumura K (2010). Urinary volatile compounds as biomarkers for lung cancer: a proof of principle study using odor signatures in mouse models of lung cancer. PLoS ONE.

[9] Lord HL, Zhan W, Pawliszyn J (2010). Fundamentals and applications of needle trap devices: a critical review. Anal. Chim. Acta.

[10] Trefz P (2012). Needle trap micro-extraction for VOC analysis: effects of packing materials and desorption parameters. J. Chromatogr. A.

[11] Rocha SM (2012). Exploring the human urine metabolomic potentialities by comprehensive two-dimensional gas chromatography coupled to time of flight mass spectrometry. J. Chromatogr. A.

[12] Monteiro M (2014). Analysis of volatile human urinary metabolome by solid-phase microextraction in combination with gas chromatography-mass spectrometry for biomarker discovery: application in a pilot study to discriminate patients with renal cell carcinoma. Eur. J. Cancer.

[13] Pawliszyn, J. *Handbook of Solid Phase Microextraction*. (Elsevier, 2011).

[14] Wercinski, S. A. *Solid phase microextraction: a practical guide*. (CRC Press, 1999).

[15] Trefz P, Rosner L, Hein D, Schubert JK, Miekisch W (2013). Evaluation of needle trap micro-extraction and automatic alveolar sampling for point-of-care breath analysis. Anal. Bioanal. Chem..

[16] Zhan W, Pawliszyn J (2012). Investigation and optimization of particle dimensions for needle trap device as an exhaustive active sampler. J. Chromatogr. A.

[17] Wishart DS (2013). HMDB 3.0–The Human Metabolome Database in 2013. Nucleic Acids Res..

[18] Schmidt K, Podmore I (2015). Current Challenges in Volatile Organic Compounds Analysis as Potential Biomarkers of Cancer. J. Biomark..

[19] Pleil JD, Stiegel MA, Risby TH (2013). Clinical breath analysis: discriminating between human endogenous compounds and exogenous (environmental) chemical confounders. J. Breath Res..

[20] Worley B, Halouska S, Powers R (2013). Utilities for quantifying separation in PCA/PLS-DA scores plots. Anal. Biochem..

[21] Worley B, Powers R (2013). Multivariate Analysis in Metabolomics. Curr Metabolomics.

[22] Xia, J. & Wishart, D. S. In *Current Protocols in Bioinformatics* (John Wiley & Sons, Inc., 2002).

[23] Deng C, Zhang X, Li N (2004). Investigation of volatile biomarkers in lung cancer blood using solid-phase microextraction and capillary gas chromatography-mass spectrometry. J. Chromatogr. B Analyt. Technol. Biomed. Life. Sci..

[24] Fuchs P, Loeseken C, Schubert JK, Miekisch W (2010). Breath gas aldehydes as biomarkers of lung cancer. Int. J. Cancer.

[25] Poli D (2010). Determination of aldehydes in exhaled breath of patients with lung cancer by means of on-fiber-derivatisation SPME-GC/MS. J. Chromatogr. B Analyt. Technol. Biomed. Life. Sci..

[26] Rudnicka J, Kowalkowski T, Ligor T, Buszewski B (2011). Determination of volatile organic compounds as biomarkers of lung cancer by SPME–GC–TOF/MS and chemometrics. J. Chromatogr. B.

[27] Buszewski B (2008). Identification of volatile organic compounds secreted from cancer tissues and bacterial cultures. J. Chromatogr. B Analyt. Technol. Biomed. Life. Sci..

[28] Hanai Y (2012). Analysis of volatile organic compounds released from human lung cancer cells and from the urine of tumor-bearing mice. Cancer Cell Int..

[29] Mieth M, Kischkel S, Schubert JK, Hein D, Miekisch W (2009). Multibed needle trap devices for on site sampling and preconcentration of volatile breath biomarkers. Anal. Chem..

