# Supplementary material for: Exploring the potential of NTME/GC-MS, in the establishment of urinary volatomic profiles. Lung cancer patients as case study
Source: Sci Rep. 2018 Aug 30;8:13113. doi: 10.1038/s41598-018-31380-y (PMC6117359; doi:10.1038/s41598-018-31380-y)
Supplement: Supplementary file 1 — Supplementary Data [file 41598_2018_31380_MOESM1_ESM.pdf]

Exploring the potential of NTME/GC-MS, in the establishment of urinary volatomic profiles. Lung cancer patients as case study

Priscilla Porto-Figueira[a], Jorge Pereira[a], Wolfram Miekisch[b], José S. Câmara[a,c,*]

[a] CQM-UMa, Centro de Química da Madeira, Universidade da Madeira, Campus Universitário da Penteada, 9020-105 Funchal, Portugal;

[b] Department of Anaesthesiology and Intensive Care Medicine, Rostock Medical Breath Research Analytics and Technologies (ROMBAT), University Medicine Rostock, Rostock, Germany.

[c] Faculdade de Ciências Exatas e da Engenharia da Universidade da Madeira, Campus Universitário da Penteada, 9020-105 Funchal, Portugal

Supplementary Figures

Supplementary Fig. 1

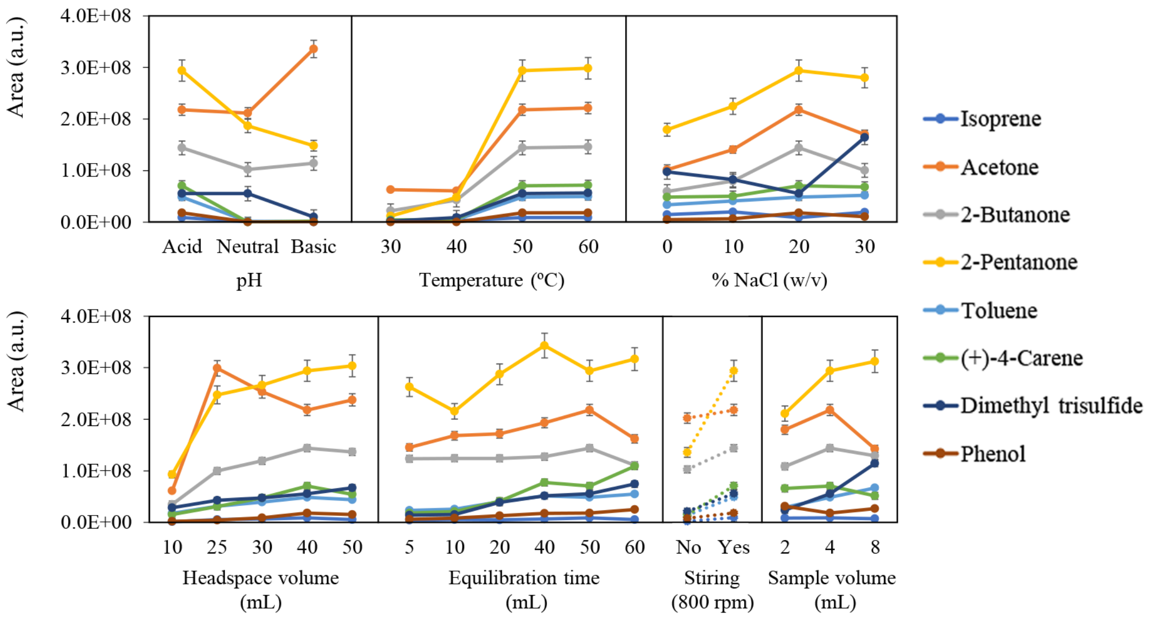

Supplementary Fig. 1: Optimization of different parameters affecting NTME (pH, extraction temperature, ionic strength through NaCl concentration, volume of the sample headspace, equilibration time, sample stirring and volume) by selected VOMs.

Supplementary Fig. 2

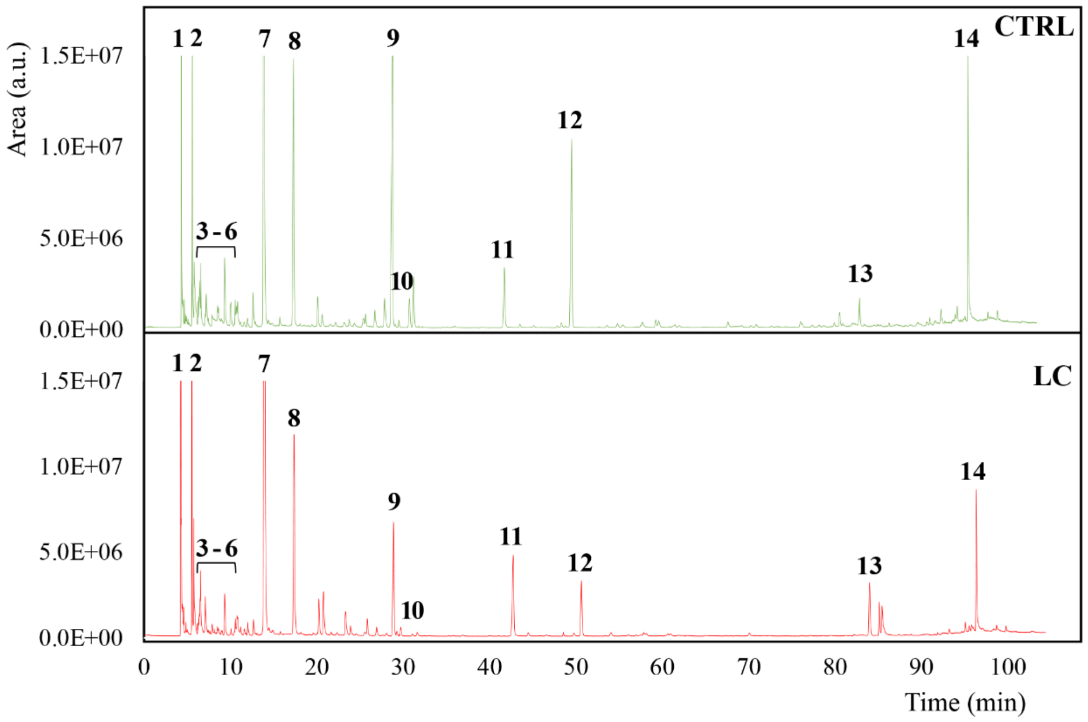

Supplementary Fig. 2: Typical chromatograms obtained with control (CTRL) and lung cancer (LC) urine samples. Legend: 1 – Furan, 2 – Acetone, 3 - Tetrahydro-2,2,5,5-tetramethylfuran, 4 - 2-Butanone, 5 - 2-Pentanone, 6 - 2,4-Dimethyl-3-pentanone, 7 - Dimethyl disulphide, 8 - 4-Heptanone, 9 - o-Cymene, 10 - methyl allyl disulphide, 11 - Dimethyl trisulfide, 12 - p-cymenene, 13 - Dehydro-Ar-ionene, 14 - p-Cresol
